# Indications for Potential Parent-of-Origin Effects within the *FTO* Gene

**DOI:** 10.1371/journal.pone.0119206

**Published:** 2015-03-20

**Authors:** Xuanshi Liu, Anke Hinney, Markus Scholz, André Scherag, Anke Tönjes, Michael Stumvoll, Peter F. Stadler, Johannes Hebebrand, Yvonne Böttcher

**Affiliations:** 1 IFB Adiposity Diseases, University of Leipzig, Leipzig, Germany; 2 Bioinformatics Group, Department of Computer Science, University of Leipzig, Leipzig, Germany; 3 Department of Child and Adolescent Psychiatry, Psychosomatics and Psychotherapy Universitätsklinikum Essen, University of Duisburg-Essen, Essen, Germany; 4 Institute for Medical Informatics, Statistics and Epidemiology, University of Leipzig, Leipzig, Germany; 5 Clinical Epidemiology, Integrated Research and Treatment Center (IFB) Center for Sepsis Control and Care (CSCC), Jena University Hospital, Jena, Germany; 6 Department of Medicine, University of Leipzig, Leipzig, Germany; 7 Fraunhofer Institute for Cell Therapy and Immunology, AG RNomics, Leipzig, Germany; 8 Interdisciplinary Center of Bioinformatics, University of Leipzig, Leipzig, Germany; 9 Institute for Theoretical Chemistry, University of Vienna, Vienna, Austria; 10 Sante Fe Institute, Santa Fe, New Mexico, United States of America

## Abstract

Genome-Wide Association Studies (GWAS) were successfully applied to discover associations with obesity. However, the GWAS design is usually based on unrelated individuals and inheritance information on the parental origin of the alleles is missing. Taking into account parent-of-origin may provide further insights into the genetic mechanisms contributing to obesity. We hypothesized that there may be variants within the robustly replicated fat mass and obesity associated (*FTO*) gene that may confer different risk for obesity depending on transmission from mother or father. Genome-wide genotypes and pedigree information from the Sorbs population were used. Phased genotypes among 525 individuals were generated by AlphaImpute. Subsequently, 22 SNPs within *FTO* introns 1 to 3 were selected and parent-of-origin specific association analyses were performed using PLINK. Interestingly, we identified several SNPs conferring different genetic effects (*P*≤0.05) depending on parental origin—among them, rs1861868, rs1121980 and rs9939973 (all in intron 1). To confirm our findings, we investigated the selected variants in 705 German trios comprising an (extremely) obese child or adolescent and both parents. Again, we observed evidence for POE effects in intron 2 and 3 (*P*≤0.05) as indicated by the parental asymmetry test. Our results suggest that the obesity risk transmitted by several *FTO* variants may depend on the parental origin of the allele. Larger family-based studies are warranted to replicate our findings.

## Introduction

Genome-Wide Association Studies (GWAS) were extremely successful in identifying novel and unexpected loci playing a role in common obesity [1]. GWAS meta-analyses revealed more than 35 loci with variants associated with BMI variation or obesity [2–6][7]. Often [7] the same variants had an impact on both BMI variation in the general population and on highly selected phenotypes such as extreme obesity with an early onset (Hinney et al. [8], Scherag et al. [9], Meyre et al. [10]). This is particularly true for the *FTO* gene which was discovered in 2007. Variants at *FTO* locus confer the largest genetic effect size among all obesity susceptibility loci and was robustly replicated in multiple populations and under various study designs [8,11–18]. In most study groups the strongest signal was reported in introns 1 or 2 (e.g. rs8050136). However, in the Sorbs, a self-contained German population, the strongest association with BMI was identified in intron 3 of *FTO* when taking into account variants from intron 1 to 3 [19]. Recent work suggests that noncoding variants in intron 1 of *FTO* may disrupt an enhancer of *IRX3* which influences the expression of *IRX3* [20,21]. However, the mechanisms of noncoding variants in intron 3 remain unclear so far.

One explanation for this discrepancy from findings in other populations might be e.g. population-specific effects in the Sorbs. Given the importance of the *FTO* locus, identifying potential POE may further improve our general understanding of the genetic mechanisms underlying obesity. For some types of cancer and Type 2 Diabetes (T2D), Kong et al [22] applied phasing algorithms to Icelandic individuals and showed considerable evidence that some genetic variants indeed confer significantly different risk depending strongly on parental origin. Recently, Hoggart et al [23] demonstrated that POE effects also exist for BMI variation based on a new method for unrelated individuals.

Moreover, despite the success of GWAS in the last couple of years still only a small proportion of the heritability can be explained by SNP variants so far, while the *FTO* variants explain ~0.34% of the inter-individual variation in BMI in European ancestry [24]. Missing inheritance information on parental origin could have resulted in a diluted marginal effect – i.e. the relatively small effect sizes identified by GWAS so far [25].

Our overall aim is to better understand if POE in Sorbs could be an explanation for the population-specific association signal in intron 3 by investigation of POE in *FTO*. Here, we tested the hypothesis that genetic variants within *FTO* confer different effects on BMI variation and obesity depending on transmission from the mother or the father. Our results suggest that several *FTO* variants may underlie parent-of-origin effects modulating the risk of obesity.

We analyzed the long-range-phased genome wide SNP data from the Sorbs population [19] in 525 individuals for whom pedigree information was available (141 families). We used the software AlphaImpute adopting the Long Range Phasing (LRP) algorithm originally developed by Kong et al. [26] which was subsequently improved by Hickey et al. [27].

We selected 22 successfully phased SNPs within *FTO* introns 1 to 3 and performed parent-of-origin specific association analyses for BMI variation. These analyses were performed using PLINK [28]. First we performed standard association tests as baseline tests using the SNPs without considering allelic inheritance. Subsequently, we used maternal and paternal alleles separately as independent variables in parent-of-origin specific association tests. To confirm our results, we analyzed 705 independent case-parent trios ascertained for early-onset obesity again focusing on parent-of-origin effects for the obesity outcome.

## Materials and Methods

### Ethical standards

All studies were approved by the ethics committees of the Universities of Leipzig or Duisburg-Essen. All subjects, or in case of minors their parents, gave written informed consent. The studies were carried out according to the *Declaration of Helsinki*.

### Individuals and pedigrees from the Sorbs population

All individuals involved in this study are part of Sorbs population (*N* = 948), a self-contained population from Eastern Germany with Slavonic origin [29][30].

The Sorbs were extensively phenotyped for a wide range of anthropometric and metabolic phenotypes including past medical and family history oGTT, T2D, weight, height, WHR and BMI as described elsewhere [19]. 525 individuals with mean age of 45.4 ± 17.2 years, mean BMI 26.1 ± 5.9 kg/m^2^ and for which pedigree information was available (141 families) were included in the present study. Identity-by-state among 525 individuals was 0.743 mirroring the degree of relatedness. 47 individuals out of 525 are affected with T2D. To avoid potential confounding we adjusted the analysis for T2D state. Characteristics of the analyzed individuals from the Sorbs population are summarized in Table 1.

**Table 1:** Characteristics of the analyzed individuals.

Sample		n total (n affected with T2D)	mean age ± SD [years]	mean BMI ± SD [kg/m^2^]	mean BMI-SDS* ± SD
Individuals for the Sorbs population	all	525 (47)	45.5 ± 17.2	26.1 ± 5.9	NA
	female	312 (31)	46.3 ± 19.4	26.0 ± 9.4	NA
	male	213 (16)	44.2 ± 20.3	26.3 ± 10.5	NA
German childhood obesity trios (adapted from Knoll et al., 2014; S1 Table)	all parents	1410	42.54 ± 6.02	30.28 ± 6.33	NA
	mothers	705	40.89 ± 5.44	32.36 ± 6.04	NA
	fathers	705	44.21 ± 6.12	31.60 ± 5.51	NA
	obese offspring	705	13.44 ± 3.01	32.02 ± 5.82	4.23 ± 1.96
	female offspring	387	13.54 ± 3.04	32.36 ± 6.04	4.50 ± 2.03
	male offspring	318	13.31 ± 2.98	31.60 ± 5.51	3.91 ± 1.81

* BMI-SDS (BMI-Standard Deviation Score) expresses is the BMI in standard deviations from a standard normal distribution relative to reference group of the same age and sex (reference data of the German National Nutrition Survey I; see Hebebrand et al. 1994 [32]).

### Childhood obesity trios from Germany

A look-up of the *FTO* SNPs was performed in a GWAS data set (genotyped by Affymetrix Genome-Wide Human SNP Array 6.0) of 705 trios comprising an extremely obese child or adolescent and both biological parents – all of central European ancestry. All index cases were at least overweight (BMI ≥90th percentile), and 83.8% were extremely obese (BMI ≥99th percentile) compared to reference data from the German National Nutrition Survey I ([31]; Table 1). Details pertaining to the phenotype and genotype quality control are reported in Knoll N et al. [31]. Characteristics of the analyzed individuals from the Childhood obesity trios are summarized in Table 1.

### Genotyping, quality control and FTO selection in the Sorbs data set

QIAmp DNA Blood Midi Kit (Qiagen Inc., Valencia, CA, USA) was used for genomic DNA extraction based on its protocol. The microarray processing and genotype calling was described in detail elsewhere [19]. We included non-imputed SNP to minimize the risk for miss-imputing using the following criteria: missing rate per SNP < 5%, Hardy–Weinberg equilibrium (HWE) P > 0.0001 and minor allele frequency (MAF) > 0.01. The average genotyping rate was about 98.7%.

In total, 387,837 SNP markers (379,772 autosomal, 8,065 X-chromosomal) overlapping from 500K Affymetrix GeneChip and the Affymetrix Genome-Wide Human SNP Array 6.0 were included in the analyses. All analyses were standardized to the forward strand. 27 *FTO*-SNPs from intron 1 to intron 3 were selected (53,737 kb to 54,879 kb according to UCSC reference genome hg19/ Genome Reference Consortium GRCh37).

### Individual re-genotyping of SNPs within FTO in the Sorbs data set

To exclude technical artifacts not captured by the quality filtering in the GWAS the variants rs8050136 and rs8053740 within *FTO* were re-genotyped in the Sorbs data set using the TaqMan SNP genotyping system (Life Technologies) according to manufacturer’s protocol. Fluorescence was detected using ABI PRISM 7500 Sequence Detecting System. To guarantee genotyping reproducibility, a random ~5% of the samples were re-genotyped in all SNPs; all genotypes matched initial designated genotypes.

### Long Range Phasing and association analysis in the Sorbs data set

The genotypes from 525 individuals were phased by the software AlphaImpute [27] using default settings. The AlphaImpute algorithm adopted the LRP [27] by integrating segregation analyses and haplotype library imputation methods. The output from AlphaImpute consists of phased haplotypes to which parental origin was assigned. Thus, AlphaImpute [27] allowed to reliable phase SNPs and to assign parent-of-origin to haplotypes. Owing to the heterozygosity of certain SNPs the phasing failed and these SNPs were excluded from further analyses. Within the *FTO* introns 1 to 3 the average phasing rate per SNP was 89%. Particularly, 5 out of 27 SNPs, showed a missing rate per SNP ≥ 30% and were removed from further analysis. Missing rates per SNP are listed in S1 Table.

To test for parent-of-origin specific associations of the 22 SNPs with BMI in the Sorbs, three different types of association tests (each 22 independent tests) were performed (Table 2). First we performed standard association tests as baseline tests using the SNPs without considering allelic inheritance. Subsequently, we used maternal and paternal alleles separately as independent variables in parent-of-origin specific association tests. These analyses were carried out using PLINK [29]. In order to perform the parent-of-origin specific association tests in PLINK, each nucleotide at each locus of the parental haplotypes was doubled to meet the analysis requirements from PLINK and to pretend maternal and paternal alleles for subsequent association analyses.

**Table 2:** Top SNPs from allelic association analyses on BMI variation in the Sorbs data set.

*FTO* location	SNP,[minor/major]*	Standard association test			Association test for parental origin						
					Paternal alleles			Maternal alleles			Student’s t-Test**
		ß[effect allele]	95% confidence interval	p-value (two-sided)	ß[effect allele]	95% confidence interval	p-value (two-sided)	ß[effect allele]	95% confidence interval	p-value (two-sided)	p-value (two-sided)
**Intron1**	rs1861869, [C/G]	0.024[G]	(0.006, 0.042)	7.29×10^–3^	0.003[G]	(-0.003, 0.010)	3.10×10^–1^	0.010[G]	(0.003, 0.016)	2.71×10^–3^	1.62×10^–1^
	rs1861868, [T/C]	-0.024[C]	(-0.005, -0.041)	8.85×10^–3^	0.003[T]	(-0.003, 0.010)	3.10×10^–1^	-0.010[C]	(-0.003, -0.017)	2.74×10^–3^	5.00×10^–3^
	rs1121980, [A/G]	-0.017[G]	(-0.035, 0.002)	7.00×10^–2^	-0.010[G]	(-0.003, -0.016)	4.86×10^–3^	0.001[G]	(-0.006, 0.007)	0.84	3.00×10^–2^
	rs9939973, [A/G]	-0.016[G]	(-0.034, 0.003)	9.00×10^–2^	-0.009[G]	(-0.002, -0.015)	9.36×10^–3^	0.001[G]	(-0.006, 0.007)	0.83	4.00×10^–2^
**Intron2**	rs10852522, [A/T]	0.023[T]	(0.004, 0.042)	2.00×10^–2^	0.003[T]	(-0.004, 0.010)	4.10×10^–1^	0.011[T]	(0.004, 0.017)	3.01×10^–3^	1.27×10^–1^
**Intron3**	rs17818920, [C/A]	0.040[A]	(0.016, 0.063)	8.10×10^–4^	0.009[A]	(0.0001, 0.017)	4.78×10^–2^	0.013[A]	(0.005, 0.022)	2.16×10^–3^	4.37×10^–1^
	rs17818902, [G/T]	0.040[T]	(0.016, 0.063)	8.29×10^–4^	0.009[T]	(0.0004, 0.017)	4.10×10^–2^	0.014[T]	(0.005, 0.022)	2.14×10^–3^	4.58×10^–1^
	rs7203051, [C/G]	0.027[G]	(0.008, 0.046)	3.98×10^–3^	0.005[G]	(-0.002, 0.012)	1.70×10^–1^	0.011[G]	(0.004, 0.017)	2.50×10^–3^	2.37×10^–1^
	rs8053740, [C/G]	0.027[G]	(0.008, 0.045)	4.47×10^–3^	0.005[G]	(-0.002, 0.012)	1.80×10^–1^	0.011[G]	(0.004, 0.017)	2.79×10^–3^	2.37×10^–1^
	rs7205009, [T/C]	0.027[C]	(0.008, 0.045)	4.84×10^–3^	0.005[C]	(-0.002, 0.011)	1.90×10^–1^	0.010[C]	(0.004, 0.017)	2.92×10^–3^	2.36×10^–1^

SNPs are presented showing evidence for association from standard, paternal, and maternal association tests adjusted by age, sex and T2D. SNPs are ordered according to the p-values of single variants per intron based on the standard association test.

*All alleles are standardized to the forward strand.

**Student´s t-Test was applied to compare linear regression derived effect estimates beta from paternal and maternal association tests and exact P values are calculated at: http://www.socscistatistics.com/pvalues/tdistribution.aspx

Prior to statistical analysis BMI was ln-transformed to approximate a normal distribution. Linear regression analysis was applied to test for allelic associations with BMI variation. All analyses were adjusted for age, sex and T2D. Student´s t-Test was applied to test for differences between linear regression derived beta values (effect size estimates) between maternal and paternal association test results. Linkage disequilibrium (r^2^) was estimated by Haploview 4.2 [33] using the Sorbs data. We applied two significance levels to our analyses. To correct for multiple testing we lowered the significance threshold to (0.05/(22*3) = 7.5x10^–4^). All *P*-values >7.5x10^–4^ but ≤ 0.05 were considered to be of nominal statistical significance. All *P*-values are provided uncorrected for multiple testing.

### Parental asymmetry test (PAT) in the childhood obesity trios from Germany

In contrast to the Sorbs data set in adults and the quantitative outcome BMI, the outcome for the childhood obesity trios from Germany was the obesity status of the index person (i.e. the offspring whose parents were afterwards ascertained). The data were analysed using the parental-asymmetry test (PAT) developed by Weinberg [34]. In short, this test focusses on heterozygous offspring and compares the number of (risk) alleles transmitted from the father/mother to all transmitted (risk) alleles using a binomial test with probability 0.5 under the null hypothesis of no parental asymmetry in the transmissions.

## Results

**Fig 1:**
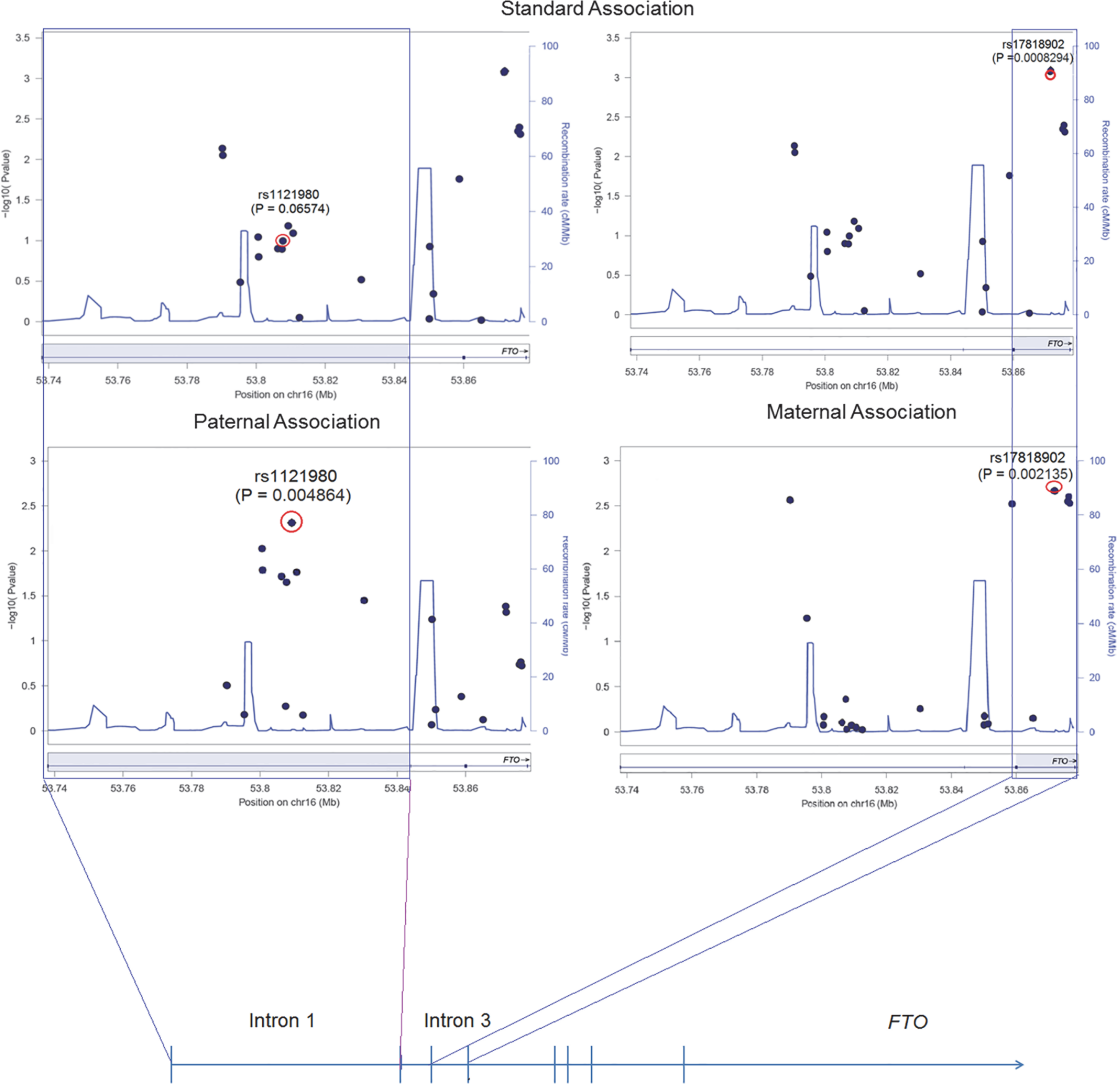
Three allelic association tests on *FTO* with BMI variation in the Sorbs data set. Different association tests are shown: top) standard association; below left) association using paternal alleles; below right) association using maternal alleles with BMI in the Sorbs adjusted for age, sex and T2D. Positions of SNPs are based on Genome Reference Consortium GRCh37. Intron 1 and 3 of *FTO* are highlighted by rectangles. Regional plots were generated by using LocusZoom version 1.1 [40].

Alleles at 10 out of 22 SNPs (mapping to introns 1, 2, and 3) showed suggestive evidence (*P* ≤ 0.05) for an association with BMI variation in either standard association test, independent maternal or paternal association tests (Fig. 1, Table 2). Individual re-genotyping of 2 variants (rs8050136, rs8053740) within *FTO* revealed reproducibility of the genotypes of 99.59% and 99.60%, respectively thus reducing the chance of technical artifacts.

### Standard association tests for BMI variation in the Sorbs data set

Using a standard association test analysis in 525 subjects out of 948 without taking into account the parent-of-origin, we identified the strongest association with BMI for two variants in intron 3 (rs17818920, rs17818902; Fig. 1, Table 2). This is in line with our previously reported GWAS on BMI variation in the Sorbs population (*N* = 948; [19]) which identified the strongest association signals not in intron 1 but intron 3 with the best association signals at rs17818902 and rs17818920.

### Parent-of-origin association tests for BMI variation in the Sorbs data set

**Fig 2:**
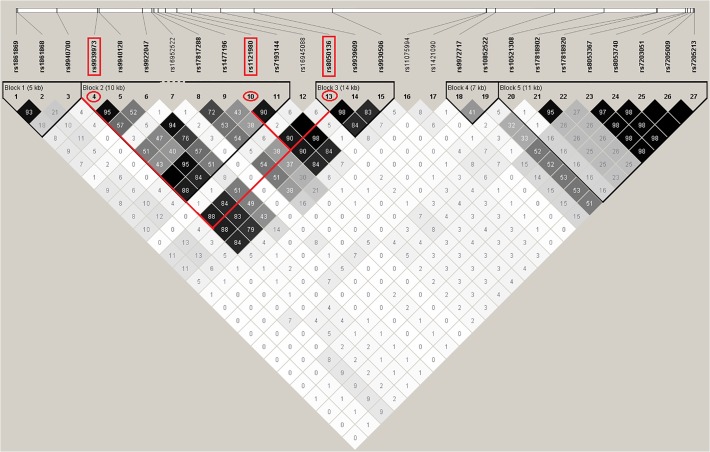
Linkage disequilibrium (LD) for all 27 SNPs in introns 1 to 3 of *FTO* in the Sorbs.

Among the 22 SNPs, 10 variants show differences in association when evaluating paternal or maternal alleles, separately. Three of the 10 variants (rs1861868, rs1121980, rs9939973; all intron 1) confer different effect directions between paternal and maternal alleles (Fig. 1, Table 2). Especially, two of them (rs1121980 and 9939973) show stronger, albeit still non-significant, relationships when considering alleles paternally transmitted compared to a standard association test setting (*P*≤0.05). Particularly, applying a Student´s t-Test comparing linear regression derived effect estimations (beta values) between paternal and maternal association tests revealed nominal differences at these SNPs (*P*≤0.05; Table 2). Interestingly, rs1121980 and rs9939973 are in high LD with rs8050136 (Fig. 2), one of the well replicated variants strongly associated with BMI (e.g. [6]). Regional plots for *FTO* comparing especially introns 1 and 3 under the different association tests are shown in Fig. 1. Among five SNPs in intron 3, we observed the smallest p-values for two variants in standard association tests. We further identified three out of five variants consistently showing indications for stronger effects in maternally transmitted alleles (Table 2). Similar results were obtained for a variant in intron 2 (rs10852522; Table 2) but the difference to paternal effects was not significant at any of these variants. In conclusion, we identified 10 *FTO* variants harboring suggestive evidence for association with BMI variation when taking into account allelic transmission from mother or father. Particularly, two of these variants within intron 1 (rs1121980, rs9939973) show, compared to association tests ignoring the parental origin, stronger effects for BMI along with evidence for differences in the effect sizes between paternal and maternal transmissions.

LD plots were generated for the Sorbs population using Haploview 4.2. Pairwise r^2^ values are shown from black (r^2^ = 1) to white (r^2^ = 0).

### Parental asymmetry tests (PAT) in the childhood obesity trios from Germany

We analyzed the 10 variants identified in the Sorbs data set in an independent data set of 705 trios comprising an extremely obese child or adolescent and both biological parents (Table 3). Albeit we do not observe evidence for POE effects for variants in intron 1 (all *P*>0.30; Table 3), we identified suggestive evidence for potential POE for variants in intron 2 and 3 (*P*≤0.05; Table 3). Notably, consistent with stronger effect sizes of maternal alleles compared to paternal alleles in intron 2 and 3 in the Sorbs we found higher transmission rates for maternal alleles in the trio data set.

**Table 3:** Top 10 SNPs (from the analyses in the Sorbs data set) analyzed for POE by PAT in the 705 German childhood obesity trios.

*FTO* location	SNP [minor/major]in Sorbs	number of paternal transmissions of the major allele to heterozygous offspring	number of maternal transmissions of the major allele to heterozygous offspring	PATp-value (two-sided)
**Intron 1**	rs1861869 [C/G]	134	119	0.38
	rs1861868 [T/C]	129	128	1.00
	rs1121980 [A/G]	140	123	0.32
	rs9939973 [A/G]	139	123	0.35
**Intron 2**	rs10852522 [A/T]	111	152	0.01
**Intron 3**	rs17818920 [C/A]	85	114	0.05
	rs17818902 [G/T]	85	114	0.05
	rs7203051 [C/G]	122	151	0.09
	rs8053740 [C/G]	122	151	0.09
	rs7205009 [T/C]	122	151	0.09

## Discussion

Despite the reported relatively large genetic effect size of *FTO *variants [12] and the general success of GWAS, large proportions of the variability in BMI cannot be explained by SNP allele variability [35]. A known shortcoming of GWAS using unrelated individuals is the limited inheritance information on the identified alleles despite recent advances to address these [23]. POE may modulate the obesity risk and might open one avenue to better understand the genetics of obesity. Recently, Hoggart et al. [23] put further weight to this hypothesis by demonstrating in unrelated individuals that POE effects on BMI exist for SNPs in *SLC2A10* and *KCNK9*. In the present study we explored POE at the *FTO* locus. We analyzed both, BMI variability in adult Sorbs and (extreme) obesity with an early onset in German trio families.

The main finding of our study is that we observed indications for potential POE at several *FTO* variants. As described earlier [19] the strongest association signals for BMI were detected in intron 3 (rs17818920 and rs17818902) involving 948 Sorbs individuals. In the present study we restricted our analyses to 525 Sorbs for whom pedigree information was available. We consistently found the strongest association results at the same two variants in intron 3 (both SNPs are in LD). Interestingly, we identified variants in intron 1 that may potentially confer different effects on BMI depending on parental origin of the transmitted allele. Several SNPs display smaller p-values when applying association tests to paternal alleles as compared to standard association tests. Our data in the Sorbs suggest that SNP alleles in intron 1 which were described to be less strongly associated to BMI than variants from intron 3 [19] may be more pronounced if the alleles are inherited from the father. This may be an indication for potential POE in the Sorbs which may be population specific given that POE effects in Sorbs have not been reported before. Particularly, alleles at rs1121980 which is in strong LD with the well-known variant rs8050136 [6] turn out to display the smallest p-values for the BMI association in the Sorbs when applying parent-of-origin specific tests. Moreover, despite the lack of evidence for difference between maternal and paternal effect sizes, we found several SNPs in intron 3 that consistently showed smaller p-values when considering maternal alleles only. Albeit not in intron 1, we also identified further indications for POE effects in introns 2 and 3 by analyzing 705 childhood obesity trios focusing the obesity outcome. These variants showed similar effect directions adding further weight to our findings in the Sorbs.

Our data may be a hint for potential effects depending on alleles inherited from the mother or the father. The Sorbs data might represent a population-specific phenomenon but may also be of broader interest as suggested by the trio data, especially for research groups working on family-based data sets. In the catalogue of POE (http://igc.otago.ac.nz/home.html) several published studies showing POE at loci on chromosome 16q are listed: e.g. for type 1 diabetes [36], bipolar affective disorder [37], psoriatic arthritis [38] and asthma [39]. A potential POE at the *FTO* locus on chromosome 16q12 may be conceivable but needs to be investigated in further, larger studies including additional functional tests and wet lab techniques such as analyzing epigenetic patterns.

However, the results of our study need to be very cautiously interpreted. None of our results withstands a correction for multiple testing. Thus, our data can only be interpreted as suggestive indications for possible POE effects in *FTO* which may be false positive. Despite our interesting explorative findings in two differently ascertained samples focusing on both BMI variation and (extreme) obesity with an early onset, our results are limited by the small sample sizes that are only powered to detect large genetic effects. Consequently, we selected the *FTO* locus with the strongest polygenic obesity signal reported so far. In sum, our data need to be cautiously interpreted and larger studies are needed to confirm our signals suggesting that POE might exist at the *FTO* locus.

## Supporting Information

S1 Table Missing rate per SNP after long range phasing. (DOC)
