# Supplementary material for: Indications for Potential Parent-of-Origin Effects within the FTO Gene
Source: PLoS One. 2015 Mar 20;10(3):e0119206. doi: 10.1371/journal.pone.0119206 (PMC4368796; doi:10.1371/journal.pone.0119206)
Supplement: S1 Table — (DOC) [file pone.0119206.s001.doc]

**S1 Table. Missing rate per SNP after long range phasing**

| **Location** | **SNP** | **Missing rate per SNP** |
| --- | --- | --- |
| **Intron 1** | rs1861869 | 7% |
|  | rs1861868 | 8% |
|  | rs9940700 | 19% |
|  | rs9939973 | 8% |
|  | rs9940128 | 8% |
|  | rs9922047 | 7% |
|  | rs16952522 | 1% |
|  | rs17817288 | 8% |
|  | **rs1477196** | **31**% |
|  | rs1121980 | 8% |
|  | rs7193144 | 9% |
|  | rs16945088 | 3% |
|  | **rs8050136** | **35**% |
|  | **rs9939609** | **35**% |
|  | rs9930506 | 8% |
| **Intron 2** | rs11075994 | 10% |
|  | rs1421090 | 5% |
|  | rs9972717 | 5% |
|  | rs10852522 | 8% |
| **Intron 3** | rs10521308 | 3% |
|  | rs17818902 | 5% |
|  | rs17818920 | 5% |
|  | **rs8053367** | **39**% |
|  | rs8053740 | 10% |
|  | rs7203051 | 10% |
|  | rs7205009 | 10% |
|  | rs7205213 | 38% |

27 SNPs are presented. Variants in bold were excluded from further analysis.
